# Ipecacuanha in Delirium Tremens

**Published:** 1858-11

**Authors:** Gerhard Paoli

**Affiliations:** Physician to the Bridewell City Prison


ARTICLE IV.

### IPECACUANHA IN DELIRIUM TREMENS.

(READ BEFORE CHICAGO MEDICAL SOCIETY.)

BY GERHARD PAOLI, M.D., PHYSICIAN TO THE BRIDEWELL CITY PRISON.

Few outside Bridewell have any idea how many of these cases almost daily arrive in a most miserable condition.

Last year I had under my treatment one hundred and sixty cases suffering with this disease, eight of which proved fatal. This year I have had up to date one hundred cases, out of whom four died. Historia morbi of many special cases might be given, but I will here limit myself to the treatment only.

Many, when they arrive, come from the most filthy and unhealthy places in the city, where they may have been lying for several days before coming to the notice of the police; half starved and dirty, covered with vermin, and many with preternaturally red noses. This will give you an idea in what situations these patients are on arriving at the famous Bridewell.

They have all for many days and weeks been constantly drinking alcohol under the name of Irish, Scotch and Bourbon, all full of poison. I know, as a fact, that this whisky contains a large quantity of fusel oil, which is a well known poison. When liquor dealers mix the above named liquors, they take large quantities of the above poison; and even brandy which is manufactured in this country is mixed with a poisoned substance which they call oil of cagmars, which is equal to Prussic acid in its effects. Knowing these facts, I wonder there are not more fatal cases.

Sleep being the suspended function, and the one nature and the system demand, we use opium to produce it, but although this medicine sometimes produces the desired effects, in many special cases it is often dangerous. Chloroform I have tried in several cases, administering it internally, as well in large as small doses, also by inhalations, with due caution; but in my experience I have not found it at all answer my expectations.

Ipecacuanha which I have tried in sixty cases, I have found most remarkably successful in the above mentioned disease; quieting the nervous system, exciting the appetite, acting on the secretions, and uniformly producing sleep. When a case is not of too long standing, I give it as an emetic the first dose, and afterwards give from fifteen to eighteen grains every other hour. Connected with this remedy, I use shower baths, and let the patient frequently drink strong beef tea, without any alcoholic stimulants.

With these facts then before us, and the experience of the happy effects of ipecacuanha, I can conscientiously recommend it to all medical practitioners.

